# Infective Endocarditis by *Clostridioides* and *Clostridium* Species—A Narrative Review

**DOI:** 10.3390/antibiotics13010033

**Published:** 2023-12-28

**Authors:** Petros Ioannou, Ioannis Kopidakis, Eirini Makraki, Stella Baliou, George Samonis

**Affiliations:** School of Medicine, University of Crete, 71003 Heraklion, Greece

**Keywords:** infective endocarditis, clostridium, clostridioides, *Clostridium septicum*, *Clostridium perfringens*

## Abstract

Bacteria of the genus *Clostridium* are anaerobic Gram-positive spore-forming bacilli that include more than 200 species. Some of them are known to cause invasive infections and diseases caused by the production of toxins. Some of the diseases that are mediated by toxins are colitis in patients with specific risk factors, such as previous administration of antimicrobials or foodborne botulism. Invasive diseases include bacteremia, infective endocarditis (IE), clostridial myonecrosis (gas gangrene), and other diseases that involve the destruction of soft tissue due to the local production of toxins. The present study aimed to review all cases of IE by *Clostridioides* and *Clostridium* species and describe the epidemiology, clinical characteristics, treatment, and outcomes of these infections. A narrative review was performed based on a search in PubMed and Scopus for studies published until 11 September 2023, providing such data of IE caused by *Clostridioides* and *Clostridium* species in humans. A total of 20 studies containing data for 21 patients were included. A prosthetic valve was present in 5 patients (23.8%). The aortic valve was the most commonly involved, followed by the mitral valve. Fever, sepsis, and embolic phenomena were the most common clinical presentations. Beta-lactams and metronidazole were the most commonly used antimicrobials. Surgery was performed in nine patients (45%). Mortality reached 33.3%. IE in multiple valves was associated with increased mortality. Despite the heterogeneous genetic and molecular characteristics that necessitate the taxonomic change of some of this genus’s previous members, the clinical syndrome of IE caused by these bacteria seems to have similar characteristics.

## 1. Introduction

Infective endocarditis (IE) is an infection of the heart, specifically of the endocardium or prosthetic material in the heart, such as prosthetic heart valves, defibrillators, pacemakers, or left ventricular assist devices, which carries significant morbidity and mortality [1,2]. IE is classically caused by aerobic Gram-positive bacteria, such as Streptococci, Staphylococci, and Enterococci. Indeed, these pathogens may add up to 75% of the isolated microorganisms in patients with IE [3,4]. IE by anaerobic bacteria is a rare disease infrequently described in the literature, primarily in case reports. Thus, the exact characteristics of IE caused by *Clostridioides* and *Clostridium* species may not be fully described [5,6].

Bacteria of the genus *Clostridium* are anaerobic Gram-positive spore-forming bacilli that include more than 200 species. Some of them are known to cause invasive infections as well as diseases caused by the production of toxins [7]. Some of the diseases that are mediated by toxins are colitis in patients with specific risk factors, such as previous administration of antimicrobials or foodborne botulism. Invasive diseases include bacteremia, IE, clostridial myonecrosis (gas gangrene), and other diseases that involve the destruction of the soft tissue due to the local production of toxins [7,8,9]. Importantly, the species *Clostridium* recently had some species reclassified to other genera, as is the case with *Clostridioides difficile*, since the genus *Clostridium* was considered phenotypically and phylogenetically incoherent [10,11]. This may have led to confusion in clinical practice, even though few infections, other than those caused by *C. difficile*, are encountered in everyday practice.

Clostridia are commonly found in the soil and may be members of the human microbiome, especially in the gastrointestinal tract [7]. These microorganisms may cause infections in people colonized by them, or sporadically, in the context of contamination of the skin after trauma [7]. Among the several different diseases caused by clostridia, *C. difficile* infection (CDI) is the most common diarrheal disease observed in hospitalized patients, usually secondary to antimicrobial exposure and alteration of the gastrointestinal microbiome, leading to the predominance of toxinogenic strains of *C. difficile* and disease through the production of enterotoxins [7,12]. Botulism, which is a relatively rare disease more commonly caused by *C. botulinum*, is associated with the production of a neurotoxin that leads to acute flaccid paralysis, with or without the development of gastrointestinal symptoms, depending on the route of the disease acquisition [7]. Tetanus is also an infrequent disease caused by the contamination and growth of *C. tetani* in puncture wounds leading to the production of a neurotoxin in people without adequate immunity, mostly in areas where vaccination against tetanus is not widespread, leading to severe central nervous system disease with muscle spasms and stiffness, often requiring intensive care unit support [7]. Food poisoning is also a disease that some clostridial species may cause. Hence, *C. perfrigens* may cause gastrointestinal disease shortly after ingesting large amount of bacteria, most commonly in the context of consuming inadequately cooked or stored food, usually meat [7]. However, other infections have been described by clostridial species, such as bacteremia, intraabdominal infections (including biliary tract infections), female genital tract infections, pleuropulmonary infections, and, rarely, IE [7,13,14,15].

IE caused by *Clostridioides* and *Clostridium* species is very rare. Evidence on this condition in the literature is mostly derived from case reports, with no systematic reviews having been published until now, and few literature reviews have been published alongside a case report [16,17].

The aim of the present study was to review all cases of IE by *Clostridioides* and *Clostridium* species and describe the epidemiology, clinical characteristics, treatment, and outcomes of these infections.

## 2. Methods

This narrative review extracted and collected data regarding *Clostridioides* and *Clostridium* spp. IE cases in humans. The primary objective of the study was to provide information on the epidemiology and mortality of these infections. The secondary outcomes were to collect and present data on (a) the exact site of infection, (b) the patients’ clinical characteristics, and (c) their treatment. For the conduction of this review, two investigators (I.K., E.M.) independently searched PubMed/Medline and Scopus databases for eligible articles reporting on “Clostrid* AND endocarditis” until 11 September 2023. Any dispute was resolved by the intervention of a senior investigator (P.I.). This narrative review included original reports on infections, such as case reports and case series that provided information on epidemiology, microbiology, treatment, and outcomes of IE by *Clostridioides* and *Clostridium* species in humans. Only articles in English were included. Reviews, systematic reviews, retrospective studies, and letters to the editor were excluded since they could not provide any original information in the synthesis of this review. Articles with no access to original data and studies referring to animal reports had to be excluded. Moreover, studies with insufficient data and articles without information on patients’ mortality and epidemiology were excluded as irrelevant. The remaining articles’ references were also searched to assess potential studies following the snowball procedure.

Two investigators (I.K., E.M.) extracted information from the eligible studies using a pre-defined template. The extracted data included study type, year of publication, and country; patients’ demographics (age and gender); patients’ relevant medical history (previous cardiac surgery or cardiac valve replacement, time after valve replacement); infection and relevant microbiology (infection site, microorganism identification, complications, and embolic phenomena); treatment administered; surgical management (if any); and outcomes (i.e., cure or death). The association of mortality with the index infection and causal microbiology was reported according to the study authors. Diagnosis of IE was confirmed by the investigators, based on information provided by the authors and the modified Duke criteria if the diagnosis was at least possible (at least 1 major and 1 minor criterion or at least 3 minor criteria) or if pathological data established a diagnosis of IE [18].

Data are presented as numbers (%) for categorical variables and median (interquartile range, IQR) for continuous variables. A univariate linear regression analysis was conducted to identify factors associated with all-cause mortality of patients. More specifically, univariate logistic regression was performed to identify any association between gender, age, presence of prosthetic cardiac valve, bad teeth hygiene or recent dental work, history of previous episode of IE, history of rheumatic heart disease, location of the infection (aortic, mitral, pulmonary, tricuspid or IE at multiple valves), presence of fever, sepsis, embolic phenomena, heart failure, antimicrobial treatment and surgical management, with all-cause mortality. Statistics were calculated with GraphPad Prism 6.0 (GraphPad Software, Inc., San Diego, CA, USA).

## 3. Results

### 3.1. Included Studies’ Characteristics 

**Figure 1:**
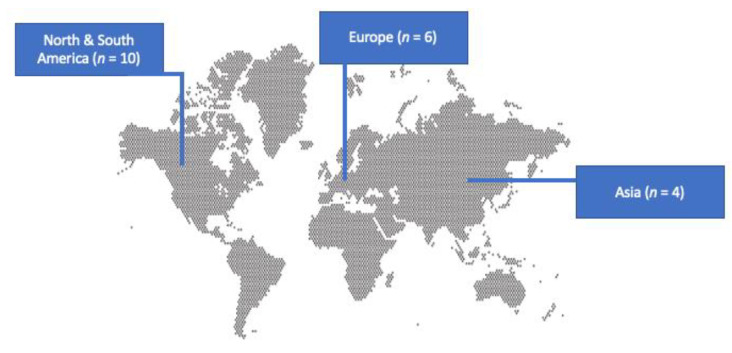
Geographical distribution of studies reporting infective endocarditis by *Clostridioides* and *Clostridium* species worldwide.

A total of 625 articles from PubMed and Scopus were screened. Of them, 20 met the present study’s inclusion criteria [16,17,19,20,21,22,23,24,25,26,27,28,29,30,31,32,33,34,35,36]. These 20 studies involved 21 patients in total. Among those studies, ten were conducted in North and South America, six in Europe, and four in Asia. There were nineteen case reports and one case series. Figure 1 shows the geographical distribution of *Clostridioides* and *Clostridium* species IE cases worldwide.

### 3.2. Epidemiology of IE by Clostridioides and Clostridium Species

The age of patients with IE by *Clostridioides* and *Clostridium* species ranged from 6 to 77 years, the median age was 31 years, and 66.7% (14 out of 21 patients) were male. Regarding predisposing factors, 23.8% (five patients) had a cardiac surgery during the three month period preceding the infection, 23.8% (five) had recently received antimicrobials, 23.8% (five) had prosthetic cardiac valve, 23.8% (five) had congenital heart disease, 14.3% (three) had history of rheumatic fever, 14.3% (three) had a previous episode of IE, 9.5% (two) had had bad oral and teeth hygiene or recent dental work, while 9.5% (two) had history of intravenous drug use (IVDU). Table 1 shows patients’ characteristics and infections’ outcomes.

### 3.3. Microbiology and Diagnosis of IE by Clostridioides and Clostridium Species

IE by *Clostridioides* and *Clostridium* species was polymicrobial in 4.8% (one patient), where, beyond *Clostridium limosum*, blood cultures were also positive for *Pseudallescheria boydii*. The isolated species from the 21 patients with IE were *C. septicum* in 19% (four patients), *C. perfrigens* in 19% (four), *C. bifermentans* in 14.3% (three), *C. difficile* in 9.5% (two), *C. sordellii* in 9.5% (two patients), *C. limosum* in 9.5% (two), *C. symbosium* in 4.8% (one), *C. inocuum* in 4.8% (one), and *C. histolyticum* in 4.8% (one). The species was not identified in 4.8% (one).

Diagnosis of IE by *Clostridioides* and *Clostridium* species was facilitated by transthoracic echocardiography in 52.4% (eleven patients), transesophageal echocardiography in 23.8% (five), autopsy in 14.3% (three), valve culture in 38.1% (nine), and foreign body culture in 4.8% (one).

### 3.4. Clinical Characteristics of IE by Clostridioides and Clostridium Species

IE by *Clostridioides* and *Clostridium* species affected the aortic valve in 45% (nine out of twenty patients with available data), the mitral valve in 35% (seven), the tricuspid valve in 10% (two), the pulmonary valve in 10% (two), a cardiac implanted electronic device (CIED) in 10% (two), and the aortic wall at the junction with a prosthetic Dacron material in 5% (one). Multiple valves were infected in 10% (two).

The most common clinical presentation included fever in 66.7% (fourteen patients), sepsis in 23.8% (five), embolic phenomena in 23.8% (five), heart failure in 19% (four), and shock in 9.5% (two).

### 3.5. Treatment and Outcomes of IE by Clostridioides and Clostridium Species

Treatment of patients with IE by *Clostridioides* and *Clostridium* species is summarized in Table 1 and detailed in Table 2. The median treatment among survivors was 5.3 weeks. The most commonly used antimicrobials were beta-lactams, and more specifically penicillin in 47.6% (10 patients), aminopenicillin in 19% (four), cephalosporin in 14.3% (three), carbapenem in 9.5% (two), and antipseudomonal penicillin in 4.8% (one). Metronidazole was administered in 47.6% (10 patients), vancomycin in 14.3% (three), aminoglycoside in 9.5% (two), linezolid in 4.8% (one), quinolone in 4.8% (one), and clindamycin in 4.8% (one). Surgical management along with antimicrobial treatment was performed in 45% (nine patients). The overall mortality was 33.3% (seven patients), and all deaths were directly attributed to the IE episode.

### 3.6. Comparison of Patients with IE by Clostridioides and Clostridium Species Who Survived with Those Who Died

Table 1 compares patients with IE by *Clostridioides* and *Clostridium* species who survived with those who died. Even though the low number of patients precluded statistical comparison, patients who survived were younger, were less likely to have had rheumatic fever and a previous IE episode, were also less likely to have IE in the aortic valve or have IE in multiple valves, fever or sepsis, and were more likely to have IE associated with a CIED.

### 3.7. Statistical Analysis of IE by Clostridioides and Clostridium Species

To identify factors associated with mortality, a univariate linear regression analysis was performed between gender, age, presence of prosthetic cardiac valve, bad oral and teeth hygiene or recent dental work, previous episode of IE, history of rheumatic heart disease, IE at the aortic, mitral, pulmonary, tricuspid valve, IE at multiple valves, presentation with fever, sepsis, embolic phenomena, development of heart failure, treatment with particular antimicrobials and presence of surgery along with antimicrobial treatment, with all-cause mortality. Among the different parameters tested, only the presence of IE by *Clostridioides* and *Clostridium* species in multiple valves was positively associated with mortality (*p* = 0.0442). The results of the linear regression analysis are shown in Table 3.

## 4. Discussion

The present study presented the characteristics of patients who developed IE by *Clostridioides* and *Clostridium* species. The most commonly intracardiac site involved was the aortic valve, followed by the mitral valve. The most common clinical presentation included fever, sepsis, embolic phenomena, and heart failure. Beta-lactams were the most commonly used treatment, with penicillin being the most frequent; however, metronidazole was also used very often. Overall mortality was 33.3%.

IE is classically caused by Gram-positive bacteria, such as staphylococci, streptococci, and enterococci [3,4]. Identification of the causative pathogen mainly relies on its isolation from blood cultures that remain the mainstay of microbiological diagnosis [37]. More specifically, receipt of blood cultures is indicated in all cases of suspected IE, with at least three sets of aerobic and anaerobic bottles per set being collected before the initiation of antimicrobial treatment [37]. Notably, even though the previous Duke criteria had specific criteria regarding the time between receipt of the blood cultures, the 2023 ISCVID-Duke criteria have removed those time requirements to simplify the diagnostic procedure [18,38]. In the case of a positive blood culture, Gram-staining helps the discrimination between Gram-positive and Gram-negative bacteria, while the shape of the bacteria and biochemical testing further allow discrimination between different species [39]. For example, Gram-positive bacteria are divided into cocci, bacilli, and those forming branches, with cocci being subdivided into catalase-negative, which include *Streptococcus* spp. and *Enterococcus* spp., and catalase-positive, including *Staphylococcus* spp. [7]. The Gram-positive bacteria that form branches are divided based on whether they are aerobic or anaerobic, with aerobic more commonly being members of the *Nocardia* species, and the anaerobic more commonly being members of the *Actinomyces* species. Gram-positive bacilli are also divided based on whether they are aerobic or anaerobic, with aerobic more commonly being members of the genus *Listeria*, *bacillus*, or *Corynebacterium*. Anaerobic Gram-positive bacteria are commonly members of the genera *Clostridium* or *Clostridioides* [7,39]. Moreover, newer molecular techniques have become popular nowadays in microbial identification, such as matrix-assisted laser desorption/ionization time-of-flight mass spectrometry (MALDI-TOF MS) or 16s rRNA gene sequencing. These two methods have allowed timely microbial identification with very high specificity even in cases where the techniques mentioned above are inconclusive [40]. More specifically, 16s rRNA PCR, combined with other molecular methods, such as next-generation sequencing, have increased the diagnostic yield in the case of IE when performed in the blood or excised valves from patients with IE [41]. These methods have now been included in the 2023 ISCVID-Duke criteria for the diagnosis of IE [18].

The present review has gathered all cases of IE by species previously considered to belong to the genus *Clostridium*. Their classification in this genus was due to their similarities. More specifically, they are Gram-positive, anaerobic, and spore-forming rods. Thus, the studies referring to these pathogens for decades used the term *Clostridium*. However, most of these species were reassigned to different genera after some taxonomic changes that occurred in recent years. These changes were necessary after studies using molecular methods revealed that this group of bacteria was quite diverse from molecular and biological perspectives [11,42]. Recent studies further support that the taxonomic changes in this genus were needed since the species included represented more than a single genus [43]. However, these taxonomic changes may lead to inconsistencies in reporting these infections, confusion to clinicians, and problems in everyday clinical practice [44].

The present patients diagnosed with IE due to *Clostridioides* and *Clostridium* species had a median age of 31 years, being lower than the age of other cohorts of patients with IE, due to other microorganisms, where the mean age is about 70 years [3,4,45]. There was a male predominance in the present patient population, being in accordance with data of IE due to other bacteria [3,45]. In the present patients, a prosthetic valve was present in 23.8%, being comparable to the corresponding rate in other studies of IE where that rate was up to 50% [3,4,45]. A previous episode of IE was noted in 14.3% of the present patients, and the same was the rate of patients with a history of rheumatic fever. Both these rates are similar in other studies in patients with IE due to other microorganisms [4,45]. Intravenous drug use was noted in 9.5% of the present patients, which is within the range noted in other studies with IE, being 4% to 9.2% [3,4,45]. Congenital heart disease was noted in 23.8% in the present study, being higher than the corresponding rate in another study reporting characteristics of IE [4].

The most commonly infected intracardiac sites were the aortic valve in 45% and the mitral in 35%. This is in line with other studies where the aortic valve was the most commonly infected, followed by the mitral valve [3,45]. As for the clinical presentation, the most common symptom was fever, which occurred in 66.7% of patients, while sepsis was evident in 23.8% and 9.5% developed shock. In other studies, fever was present in 84% [4], and shock was diagnosed in 9% of patients [3]. Heart failure was diagnosed in 19% of the present patients, which is slightly lower than the proportion reported by other studies that ranged from 33% to 52% [3,45]. Embolic phenomena in the present study were present in 19%, which is a rate similar to that noted in other studies in the general population, where the rate ranged from 15% to 45% [3,4].

Regarding treatment of the present patients, beta-lactams and metronidazole were the most common regimens used. This aligns with the literature where studies support that most clostridial species retain sensitivity to beta-lactams [46,47,48,49,50,51,52]. At the same time, metronidazole is also a potent antimicrobial, given the anaerobic nature of these pathogens and the potent activity of this medication to this class of microorganisms [53,54]. However, the results of this study should be read with caution, since the group of pathogens described herein is not homogenous; thus, a general conclusion regarding the suitability of antimicrobials cannot be drawn with applicability to all species. For example, resistance to metronidazole is relatively common for *C. difficile* and increasingly recognized in *C. perfrigens* as well [55,56].

In the present review, in-hospital mortality was 33.3%, and all patients died due to their infection. This rate was comparable to that noted in other studies, where mortality was within the range of 11–40% [3,4,45]. Importantly, the present finding identified IE in multiple valves associated with increased mortality.

This study has some notable limitations. First, it mainly consists of a small number of case reports; thus, the evidence may be low. However, given the rarity of the cases, it is unclear whether a prospective or retrospective study could provide an adequate number of cases, even if it included cases over many years and from many participating centers. The small number of cases in the present review precluded a detailed statistical analysis, such as the Mann–Whitney test or chi-square test, among patients who died and those who survived. Moreover, this study is a narrative, not a systematic review; thus, the evidence provided is limited, even if statistical analysis was conducted. Additionally, due to the inconsistency of the classification of the causal bacteria in the genus *Clostridium*, including some studies in the present review may sound controversial to some experts. However, we felt that including all studies on IE by *Clostridioides* and *Clostridium* species, based on the classification that was valid until some months ago, would simplify the conduction of this review. Finally, due to a possible heterogeneity in the studies, mostly relating to the large time among the different studies, differences in the diagnosis and the microbiological assays used for pathogen identification may have influenced the results of the present study.

## 5. Conclusions

To conclude, this narrative review updates the information on IE by *Clostridioides* and *Clostridium* species, by comprehensively describing the epidemiology, clinical characteristics, treatment, and outcomes of this infection, and provides a detailed comparison of these characteristics with those of IE by other species. Beta-lactams and metronidazole were the most commonly used antimicrobials for treatment. Infection of multiple heart valves was associated with mortality. Despite the heterogeneous genetic and molecular characteristics that necessitate the taxonomic change of some of this genus’s previous members, the clinical syndrome of IE caused by these bacteria seems to have similar characteristics.

## Figures and Tables

**Table 1:** Patients’ characteristics and infections’ outcome.

Characteristic	All Patients (*n* = 21) *	Survived (*n* = 14)	Died (*n* = 7)
Age, years, median (IQR)	31 (21–60)	27 (19.5–62.3)	52 (23–59)
Male gender, *n* (%)	14 (66.7)	10 (71.4)	4 (57.1)
Predisposing factors			
Post cardiac surgery, *n* (%)	5 (23.8)	3 (21.4)	2 (28.6)
Previously on antibiotics, *n* (%)	5 (23.8)	3 (21.4)	2 (28.6)
Congenital heart disease, *n* (%)	5 (23.8)	4 (28.6)	1 (14.3)
Prosthetic valve, *n* (%)	5 (23.8)	3 (21.4)	2 (28.6)
Rheumatic fever, *n* (%)	3 (14.3)	1 (7.1)	2 (28.6)
Previous IE, *n* (%)	3 (14.3)	1 (7.1)	2 (28.6)
IVDU, *n* (%)	2 (9.5)	2 (14.3)	0 (0)
Bad teeth hygiene or recent dental work, *n* (%)	2 (9.5)	2 (14.3)	0 (0)
Method of diagnosis			
Transthoracic echocardiography, *n* (%)	11 (52.4)	9 (64.3)	2 (28.6)
Transesophageal echocardiography, *n* (%)	5 (23.8)	3 (21.4)	2 (28.6)
Autopsy, *n* (%)	3 (14.3)	0 (0)	3 (42.9)
Valve culture, *n* (%)	8 (38.1)	6 (42.9)	2 (28.6)
Valve localization			
Aortic valve, *n* (%)	9 out of 20 (45)	4 out of 13 (30.8)	5 (71.4)
Mitral valve, *n* (%)	7 out of 20 (35)	5 out of 13 (38.5)	2 (28.6)
Tricuspid valve, *n* (%)	2 out of 20 (10)	1 out of 13 (7.7)	1 (14.3)
Pulmonary valve, *n* (%)	2 out of 20 (10)	1 out of 13 (7.7)	1 (14.3)
Multiple valves, *n* (%)	2 out of 20 (10)	0 out of 13 (0)	2 (28.6)
CIED, *n* (%)	2 out of 20 (10)	2 out of 13 (15.4)	0 (0)
Other **, *n* (%)	1 out of 20 (5)	1 out of 13 (7.7)	0 (0)
Clinical characteristics			
Fever, *n* (%)	14 (66.7)	8 (57.1)	6 (85.7)
Sepsis, *n* (%)	5 (23.8)	2 (14.3)	3 (42.9)
Heart failure, *n* (%)	4 (19)	3 (21.4)	1 (14.3)
Embolic phenomena, *n* (%)	5 (23.8)	2 (14.3)	3 (42.9)
Shock, *n* (%)	2 (9.5)	1 (7.1)	1 (14.3)
Treatment			
Duration of treatment in weeks, median (IQR)	NA	5.3 (3.3–6)	NA
Metronidazole, *n* (%)	10 (47.6)	7 (50)	3 (42.9)
Penicillin, *n* (%)	10 (47.6)	7 (50)	3 (42.9)
Aminopenicillin, *n* (%)	4 (19)	3 (21.4)	1 (14.3)
Vancomycin, *n* (%)	3 (14.3)	3 (21.4)	0 (0)
Cephalosporin, *n* (%)	3 (14.3)	2 (14.3)	1 (14.3)
Carbapenem, *n* (%)	2 (9.5)	2 (14.3)	0 (0)
Aminoglycoside, *n* (%)	2 (9.5)	1 (7.1)	1 (14.3)
Linezolid, *n* (%)	1 (4.8)	1 (7.1)	0 (0)
Quinolone, *n* (%)	1 (4.8)	1 (7.1)	0 (0)
Clindamycin, *n* (%)	1 (4.8)	0 (0)	1 (14.3)
Antipseudomonal penicillin, *n* (%)	1 (4.8)	0 (0)	1 (14.3)
Surgical management, *n* (%)	9 out of 20 (45)	6 out of 13 (46.2)	3 (42.9)
Outcomes			
Deaths due to infection, *n* (%)	7 (33.3)	NA	NA
Deaths overall, *n* (%)	7 (33.3)	NA	NA

CIED: cardiac implanted electronic device; IE: infective endocarditis; IQR: interquartile range; IVDU: intravenous drug use; NA: not applicable; *: data are among the number of patients mentioned on top unless otherwise described; **: infection at the junction of the aortic wall with the Dacron prosthesis in one case.

**Table 2:** Characteristics of the included studies.

Study	Number of Patients	Age (Years)	Gender	Site of Infection *n* (%)	Microbiology of Infection, *n* (%)	Treatment Administered, *n* (%)	Infection Outcomes, *n* (%)
More et al., 1943 [19]	1	34	Female	AoV 1 (100)	*C. perfrigens* (*welchii*) 1 (100)	Sulphanilamide 1 (100)	Clinical cure ^a^ 0 (0) Deaths overall 1 (100) Deaths due to IE 1 (100)
Alvarez-Elcoro et al., 1984 [20]	1	23	Male	AoV 1 (100) MV 1 (100)	*C. perfringens* 1 (100)	Penicillin 1 (100)	Clinical cure 0 (0) Deaths overall 1 (100)Deaths due to IE 1 (100)
Gordon et al., 1985 [21]	1	52	Male	AoV 1 (100)	*C. limosum* 1 (100)	Penicillin 1 (100) Aminoglycoside 1 (100) Metronidazole 1 (100) Surgical management 1 (100)	Clinical cure 1 (100) Deaths overall 1 (100) Deaths due to IE 0 (0)
Barnes et al., 1987 [22]	1	61	Male	MV 1 (100)	*C. sordelli* 1 (100)	Penicillin 1 (100)	Clinical cure 1 (100) Deaths overall 0 (0)
Kolander et al., 1989 [16]	1	23	Male	MV 1 (100)	*C. bifermentans* 1 (100)	Penicillin 1 (100) Metronidazole 1 (100)	Clinical cure 1 (100) Deaths overall 0 (0)
Ridgway et al., 1993 [17]	1	59	Female	MV 1 (100)	*C. septicum* 1 (100)	Penicillin 1 (100) Aminopenicillin 1 (100) Metronidazole 1 (100) Surgical management 1 (100)	Clinical cure 0 (0) Deaths overall 1 (100) Deaths due to IE 1 (100)
Moyano et al., 1994 [23]	1	26	Male	TrV 1 (100)	*C. bifermentans* 1 (100)	Penicillin 1 (100)	Clinical cure 1 (100) Deaths overall 0 (0)
Cutrona et al., 1995 [24]	1	18	Female	PV 1 (100) TrV 1 (100)	*C. innocuum* 1 (100)		Clinical cure 0 (0) Deaths overall 1 (100) Deaths due to IE 1 (100)
Mendes et al., 1996 [25]	1	31	Male	AoV and aortic wall at Dacron prosthesis 1 (100)	*C. perfringens* 1 (100)	Penicillin 1 (100)	Clinical cure 1 (100) Deaths overall 0 (0)
Holland et al., 1997 [26]	1	20	Male	MV 1 (100)	*Clostridium* spp. 1 (100)	Cephalosporin 1 (100)	Clinical cure 1 (100) Deaths overall 0 (0)
Cohen et al., 1998 [27]	1	77	Male	AoV 1 (100)	*C. septicum* 1 (100)	Cephalosporin 1 (100) Metronidazole 1 (100)	Clinical cure 0 (0) Deaths overall 1 (100) Deaths due to IE 1 (100)
Fisker et al., 1998 [28]	1	6	Male	PV 1 (100)	*C. septicum* 1 (100)	Penicillin 1 (100) Surgical management 1 (100)	Clinical cure 1 (100) Deaths overall 0 (0)
Koch et al., 1999 [29]	2	77, 66	1 Female, 1 Male	CIED 1 (50) MV 1 (50)	*C. perfringens* 1 (50) *C. symbosium* 1 (50)	Penicillin 1 (50) Aminopenicillin 1 (50) Metronidazole 1 (50)	Clinical cure 2 (100) Deaths overall 0 (0)
Durmaz et al., 2000 [30]	1	18	Female	AoV 1 (100)	*C. histolyticum* 1 (100)	Penicillin 1 (100) Aminoglycoside 1 (100) Metronidazole 1 (100) Surgical management 1 (100)	Clinical cure 1 (100) Deaths overall 0 (0)
Chaudhry et al., 2014 [31]	1	22	Male	MV 1 (100)	*C. bifermentans* 1 (100)	Carbapenem 1 (100) Vancomycin 1 (100) Linezolid 1 (100) Quinolone 1 (100) Metronidazole 1 (100)	Clinical cure 1 (100) Deaths overall 0 (0)
Yung et al., 2019 [32]	1	59	Male	AoV 1 (100)	*C. limosum* 1 (100)	Aminopenicillin 1 (100) Cephalosporin 1 (100)Carbapenem 1 (100) Vancomycin 1 (100)Metronidazole 1 (100) Surgical management 1 (100)	Clinical cure 1 (100) Deaths overall 0 (0)
Berkefeld et al., 2020 [33]	1	75	Male	CIED 1 (100)	*C. difficile* 1 (100)	Aminopenicillin 1 (100) Vancomycin 1 (100) Metronidazole 1 (100) Surgical management 1 (100)	Clinical cure 1 (100) Deaths overall 0 (0)
Chaudhry et al., 2021 [34]	1	11	Female	NR 1 (100)	*C. difficile* 1 (100)	Metronidazole 1 (100) Surgical management 1 (100)	Clinical cure 1 (100) Deaths overall 0 (0)
Chaudhry et al., 2021 [35]	1	28	Female	AoV 1 (100)	*C. sordelli* 1 (100)	Metronidazole 1 (100) Surgical management 1 (100)	Clinical cure 1 (100) Deaths overall 0 (0)
Dubois et al., 2022 [36]	1	58	Male	AoV 1 (100)	*C. septicum* 1 (100)	Antipseudomonal penicillin 1 (100) Clindamycin 1 (100) Surgical management 1 (100)	Clinical cure 0 (0) Deaths overall 1 (100) Deaths due to IE 1 (100)

^a^ Defined as the clinical resolution of the infection as a result of treatment. AoV: aortic valve; CIED: cardiac implantable electronic device; MV: mitral valve; PV: pulmonary valve TrV: tricuspid valve.

**Table 3:** Univariate linear regression analysis of patients with infective endocarditis by *Clostridioides* and *Clostridium* species between different parameters and overall mortality.

Characteristic	Univariate Analysis *p*-Value
Age	0.4477
Male gender	0.5367
Prosthetic cardiac valve	0.7334
Bad oral and teeth hygiene or recent dental work	0.3171
Previous episode of IE	0.2044
History of rheumatic heart disease	0.2044
IE in the aortic valve	0.0893
IE in the mitral valve	0.6783
IE in the pulmonary valve	0.6601
IE in the tricuspid valve	0.6601
IE in multiple valves	0.0442
Fever	0.0293
Sepsis	0.1626
Embolic phenomena	0.1626
Heart failure	0.7117
Surgical management	0.8948
Penicillin	0.7715
Ampicillin	0.7117
Metronidazole	0.7715

IE: infective endocarditis.
